# Versatile ion S5XL sequencer for targeted next generation sequencing of solid tumors in a clinical laboratory

**DOI:** 10.1371/journal.pone.0181968

**Published:** 2017-08-02

**Authors:** Meenakshi Mehrotra, Dzifa Yawa Duose, Rajesh R. Singh, Bedia A. Barkoh, Jawad Manekia, Michael A. Harmon, Keyur P. Patel, Mark J. Routbort, L. Jeffrey Medeiros, Ignacio I. Wistuba, Rajyalakshmi Luthra

**Affiliations:** 1 Department of Hematopathology, The University of Texas MD Anderson Cancer Center, Houston, TX, United States of America; 2 Department of Translational Molecular Pathology, The University of Texas MD Anderson Cancer Center, Houston, TX, United States of America; Mayo Clinic Arizona, UNITED STATES

## Abstract

**Background:**

Next generation sequencing based tumor tissue genotyping involves complex workflow and a relatively longer turnaround time. Semiconductor based next generation platforms varied from low throughput Ion PGM to high throughput Ion Proton and Ion S5XL sequencer. In this study, we compared Ion PGM and Ion Proton, with a new Ion S5XL NGS system for workflow scalability, analytical sensitivity and specificity, turnaround time and sequencing performance in a clinical laboratory.

**Methods:**

Eighteen solid tumor samples positive for various mutations as detected previously by Ion PGM and Ion Proton were selected for study. Libraries were prepared using DNA (range10-40ng) from micro-dissected formalin-fixed, paraffin-embedded (FFPE) specimens using the Ion Ampliseq Library Kit 2.0 for comprehensive cancer (CCP), oncomine comprehensive cancer (OCP) and cancer hotspot panel v2 (CHPv2) panel as per manufacturer’s instructions. The CHPv2 were sequenced using Ion PGM whereas CCP and OCP were sequenced using Ion Proton respectively. All the three libraries were further sequenced individually (S540) or multiplexed (S530) using Ion S5XL. For S5XL, Ion chef was used to automate template preparation, enrichment of ion spheres and chip loading. Data analysis was performed using Torrent Suite 4.6 software on board S5XL and Ion Reporter. A limit of detection and reproducibility studies was performed using serially diluted DLD1 cell line.

**Results:**

A total of 241 variant calls (235 single nucleotide variants and 6 indels) expected in the studied cohort were successfully detected by S5XL with 100% and 97% concordance with Ion PGM and Proton, respectively. Sequencing run time was reduced from 4.5 to 2.5 hours with output range of 3–5 GB (S530) and 8–9.3Gb (S540). Data analysis time for the Ion S5XL is faster 1 h (S520), 2.5 h (S530) and 5 h (S540) chip, respectively as compared to the Ion PGM (3.5–5 h) and Ion Proton (8h). A limit detection of 5% allelic frequency was established along with high inter-run reproducibility.

**Conclusion:**

Ion S5XL system simplified workflow in a clinical laboratory, was feasible for running smaller and larger panels on the same instrument, had a shorter turnaround time, and showed good concordance for variant calls with similar sensitivity and reproducibility as the Ion PGM and Proton.

## Introduction

Genomic alterations in cancer are important biomarkers and can be used to facilitate prognostic stratification and guide the selection of targeted therapies [1–3]. Several molecular testing platforms are currently available for genotyping somatic mutations. With an increase in demand for clinical screening of multiple markers, traditional polymerase chain reaction (PCR)- based single gene sequencing using sanger sequencing, pyro sequencing and capillary electrophoresis has become difficult, owing to the increasing sample requirement, low sequencing throughput and need for maintaining multiple testing platforms [1, 4–7].

Currently, next generation sequencing-based molecular profiling of tumors is an important part of clinical practice for the management of cancer patients owing to the ability of NGS to simultaneously screen for a variety of genomic aberrations in multiple patients using a one-time relatively low input of nucleic acid [1, 8]. Although whole-genome sequencing offers the most complete strategy for tumor analysis, its present utility in clinical care is limited [9]. Instead, targeted sequencing of hundreds of genes of established clinical significance at deeper sequencing depths can facilitate increased detection sensitivity for mutations in heterogeneous or low purity tumors [10–12]. However, there are challenges in establishing a clinical NGS workflow including tumor specimen acquisition and quality, tumor heterogeneity, incidental germline mutations, scalability, cost, turnaround time, and clinical interpretation of results [13–15].

NGS technologies are rapidly evolving with time. The choice for a NGS-based genotyping platform is mainly a trade-off between rapidity of obtaining results and the amount of genetic information that is delivered in a rapid manner [5, 16]. In this regard semiconductor–based Ion Torrent sequencing technology has gained considerable acceptance in clinical diagnostic laboratories due to the small size of the sequencer, low DNA input requirement, compatibility with FFPE derived DNA and availability of useful panels including a small cancer hotspot panel V2 (CHPv2 250 amplicons), an intermediate the Oncomine comprehensive cancer panel (OCP 2,530 amplicons) and a large comprehensive cancer panel (CCP 16,000 amplicons), with short turnaround time (2.5–4 hours per run) for sequencing [5, 16, 17]. Ion Torrent sequencing is performed in microscopic wells interfaced with a semiconductor chip. DNA of interest is clonally amplified on microscopic beads and unlabeled nucleotides are introduced in a predetermined order, one at a time. Upon incorporation, protons that are released from the 3’-OH group during formation of the phosphodiester bonds result in a change in pH, which is measured by the semiconductor chip. Among different types of Ion Torrent sequencers, the Ion PGM is the most popular sequencer although it has the limitation of being capable of sequencing only smaller panels (screening ≤ 50 genes; 12 samples per run) or an intermediate oncomine panel (1sample per run). The Ion Proton sequencer allows sequencing of larger panels, such as the OCP or CCP, but due to high-capacity it is cost prohibitive to be used routinely for smaller gene panels like CHPv2, unless large batches of multiplexed samples are sequenced.

Implementing NGS assays in the clinic for the routine screening of cancer patients requires rigorous validation in a College of American Pathologists certified and Clinical Laboratory Improvement Amendement accredited laboratory which includes establishment of recommended performance parameters to insure consistent and reliable performance of a test on FFPE samples with high sensitivity and specificity [18, 19]. Consequently, for small scale laboratories, it may be difficult and cost prohibitive to maintain the validation and maintenance of different capacity sequencers to accommodate sequencing of gene panels of different sizes. Ion S5XL provides the much needed flexibility to run both smaller and larger panels according to laboratory needs. The Ion S5XL offers sequencing at high speeds and at a low cost per base with deeper coverage.

In this study, we compared different Ion Torrent NGS platforms including the Ion PGM, Ion Proton and Ion S5XL for mutational analysis using Ion Ampliseq Panels of different sizes, namely a comprehensive cancer panel (CCP 16,000 amplicons), Oncomine comprehensive cancer panel (OCP 2,530 amplicons) and cancer hotspot panelv2 (CHPv2 250 amplicons) in a pre-clinical setting. We focused on detection sensitivity, mutation specificity, and capacity, ease of use and speed of sequencing.

## Materials and methods

### Tumor samples

The study group included 18 solid tumor and normal paired samples from diverse advance cancers including: 12 carcinomas, 3 brain tumors, 2 melanoma and 1 sarcoma. The carcinomas were derived from the lung (n = 3), colon (n = 3), kidney (n = 2), endometrium (n = 1), and liver (n = 1). The age of the FFPE tissue biopsy samples retrieved from storage ranged from 1 to 8 years. This study protocol was approved by the Institutional Review Board of MD Anderson Cancer Center and is consistent with international ethical standards on human subject research. The tumor samples were known to be positive for various mutations using the semiconductor-based Ion PGM and Ion Proton sequencer performed previously in a CLIA-certified molecular diagnostic laboratory. The cell lines used included DLD1 (ATCC-CCL221), and HL60 (ATCC-CCL240). Cell line pellets were fixed in formalin and embedded in paraffin to mimic routinely processed tissue biopsy specimens.

### Tissue selection and DNA extraction

Hematoxylin and eosin stained tissue sections of tumor biopsy samples were reviewed by a pathologist who circled the tumor area and estimated tumor cellularity in a circled area. Specimens with a minimum of 20% tumor in the circled area were selected for this study. DNA was extracted from FFPE tumor samples using a PicoPure DNA extraction kit (Arcturus, Mountain View, CA) and purified using the Agencourt AMPure XP kit (Agencourt Biosciences, Beverly, MA). Qubit DNA high-sensitivity assay kit (Life Technologies, Carlsbad, CA) was used to quantify purified DNA.

### Ion Ampliseq targeted panels selected for study

Ion Ampliseq targeted CHPv2; OCP and CCP panels were included in the study. The Ion Ampliseq Cancer Hot spot panel V2 (CHPv2 250 amplicons) (ThermoFisher Scientific, Waltham, MA) covers mutations in hotspot regions of 50 genes in one prime pool and requires 10ng of genomic DNA for library preparation and sequencing on the Ion PGM sequencer (Life Technologies). The Oncomine Cancer Panel (OCP) that includes a total of 143 genes of which 73 oncogenes are interrogated for mutational hotspots and 26 tumor suppressor genes are interrogated for all exons. In addition, the OCP provides the capability of detecting copy number variations in 49 genes and fusion drivers in 22 genes. The DNA part of this panel is composed of two multiplexed primer pools and requires a total of 20 ng of DNA template for each sample (10 ng per pool). The Comprehensive Cancer Panel (CCP) is a relatively large panel designed to facilitate amplification-based capture and sequencing of entire coding regions of 409 cancer-related genes. This panel includes four primer pools with approximately 4,000 primer pairs in each pool and requires a total of 40 ng of DNA as a template for each sample (10 ng per pool).

### Sequencing on Ion PGM platform

**Fig 1:**
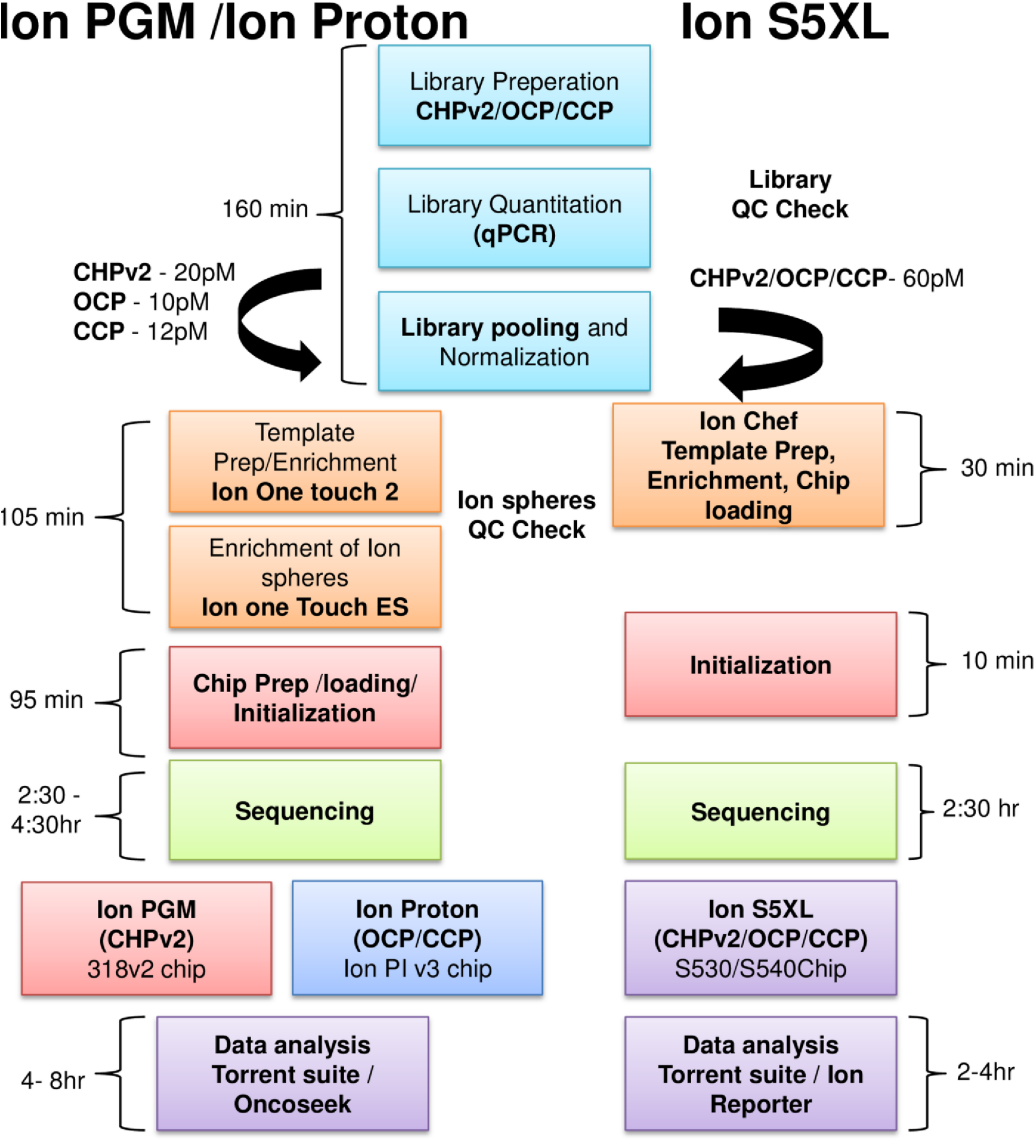
NGS workflow used for different Ion Torrent platforms in a clinical laboratory. Ion PGM, Ion Proton and Ion S5XL sequencing workflow using cancer hot spot panel v2 (CHPv2), oncomine panel (OCP) and comprehensive cancer panel (CCP) for library prep. Ion one touch 2 and Ion one touch ES was used for PGM and Proton for template preparation and enrichment of ion spheres. Enriched ion spheres were sequenced on Ion PGM and Ion Proton respectively. For Ion S5XL Ion chef was used which automates the template preparation, enrichment and chip loading. Preloaded reagent cartridge and on board Torrent suite software reduces the initialization, sequencing and data analysis time.

For sequencing on the Ion PGM platform (ThermoFisher Scientific), libraries were prepared from 10 ng tumor DNA by using the Ion Torrent Ampliseq 2.0 kit (ThermoFisher Scientific) and CHPv2 panel according to the manufacturer's instructions. Samples were barcoded and quantified by qPCR using the Ion Xpress Barcode Adapter 1–96 kit and the Ion Library Taqman quantitation kit (ThermoFisher Scientific), respectively. Libraries from 12 samples were pooled at 20 pM concentrations for sequencing on the Ion PGM 318 v2 chip. Pooled libraries were clonally amplified on Ion Spheres (ThermoFisher Scientific). Emulsion PCR (e-PCR) was performed using the Ion PGM Template OT2 kit v2 and the Ion One Touch 2 system (ThermoFisher Scientific) as per manufacturer's instructions. Ion Sphere particles (ISPs) were enriched by using the Ion Torrent OneTouch ES (ThermoFisher Scientific). Enriched ISPs were loaded on a PGM 318 v2 chip and sequenced on the Ion PGM platform by using the Ion PGM sequencing kit (Fig 1).

### Sequencing on Ion Proton platform

For sequencing on the Ion Proton platform (ThermoFisher Scientific), libraries were prepared from 20 and 40 ng tumor DNA, by using the OCP or CCP panel, respectively and the Ion Torrent Ampliseq 2.0 kit (ThermoFisher Scientific) according to the manufacturer's instructions. Samples were barcoded and libraries were prepared. The 12 pM pooled libraries were clonally amplified and e-PCR was performed by using the Ion PI HI-Q Template OT2 kit and Ion One Touch 2 system (ThermoFisher Scientific) according to the manufacturer's instructions. Pooled libraries from 5 and 12 normal tumor-paired samples were sequenced per run for CCP and OCP, respectively on the Ion Proton using the Ion HI-Q PI Chip v3 and Ion PI HI-Q sequencing 200 kit (ThermoFisher Scientific) (Fig 1).

### Sequencing on Ion S5XL platform

The CHPv2, CCP and OCP libraries sequenced on the Ion PGM and Proton were used for validation of the Ion S5XL and compared for mutation detection sensitivity, specificity, scalability, costs and turnaround time. A 60pM pooled library from these different panels was used for template preparation on the Ion Chef and subsequently sequenced on S5XL using Ion S530 and S540 chips as per manufacturer’s instruction. Libraries from the three different panels were pooled together in equimolar ratio (60pM) and multiplexed (S530 chip) or individually (S540 chip) sequenced (Fig 1 and S1 Table). The size of a targeted gene panel tested decides the level of multiplexing on a chip for sequencing (large panels have low levels of multiplexing and small panels have high levels). A sequencing coverage of 250X, variant coverage of 25X and variant allelic frequency of 5% were set as cutoffs to avoid any false positive and false negative results.

### Data analysis pipeline for Ion PGM, Proton and S5XL

Analysis of the raw sequencing data and alignment of sequencing reads to the reference genome (Human genome build 19 of Hg 19) was performed by the Ion Torrent Suite software v4.4.2 for Ion PGM and Ion Proton (ThermoFisher Scientific). Identification of sequence variants was performed by Ion Torrent Variant Caller Plugin software v4.4-r76860, and coverage of each amplicon was determined by the Coverage Analysis Plugin software v4.4-r77897. Custom software developed in-house (OncoSeek) was used to interface the data generated by the Ion Torrent Variant Caller with the IGV, filter repeat errors due to nucleotide homopolymer regions, compare replicate samples, and annotate the sequencing information [18]. Integrative Genome Viewer (IGV) was used to visualize the read alignment and the presence of variants against the reference genome as well as to confirm variant calls by checking for strand biases and sequencing errors. A minimum sequencing depth of 250X was used as a measure of successful sequencing of a sample. For Ion S5XL primary and secondary analysis was performed using Torrent Suite 4.6 (on board Ion S5XL) and Ion Reporter software, respectively. Analysis was compared for speed of result output, sequencing quality metrics; mutation allelic frequencies observed on Ion S5XL were compared to those identified on Ion PGM and Ion Proton to establish concordance.

### Limit-of-detection analysis

The mutation detection sensitivity of Ion S5XL was determined by using DLD1 cell line DNA positive for heterozygous mutations in six different genes: *PIK3CA*, *KRAS*, *KIT*, *TP53*, *FGFR1* and *SMO* as a wild type cell line, HL60. DLD1 DNA was serially diluted with HL60 to obtain samples with 50%, 25%, 12.5%, 6.25%, 3.15%, 1.5%, or 0.75% positive (mutated) DNA in wild-type DNA. Samples were tagged with different barcodes and sequenced on S530 chip on Ion S5XL.

### Inter run reproducibility study

Inter-run reproducibility studies were performed by sequencing one normal tumor paired CCP and one OCP libraries prepared from a patient DNA sample indexed with different barcodes that were multiplexed and sequenced twice on a different S530 chip on Ion S5XL.

## Results

### Patient samples and cohort summary

Normal tumor paired FFPE samples were collected from 18 patients with advanced cancer of various types and known tumor mutation status as detected analysis using the Ion PGM and Ion Proton. The median age for the patient cohort was 59 years (range, 20–76 years), and there were 9 men and 9 women. At last follow up, 9 (50%) patients were alive and 9 (50%) had died. Mutations (n = 241) in a number of genes were identified in the tumor tissues using the Ion Ampliseq Cancer Hotspot Panel V2 on the Ion PGM platform and the OCP and CCP on Ion Proton were compared. The median number of altered mutations per tumor was 13 (range, 2–71).

### Workflow advantages of S5XL

Ion S5XL workflow utilizes Ion Chef which provides an automation for template preparation, enrichment of template ion spheres and template loading on Ion chip which reduces the labor time and manual errors (Fig 1). Availability of different S5XL ion chips provides scalability to use different panels on the same instrument according to laboratory needs. Hence, Ion S5XL eliminates the need for keeping two different sequencing capacity Ion Torrent instruments (PGM and Proton) and saves technical and annual maintenance costs. The Ion S5XL instrument requires less maintenance as compared to Ion PGM and Ion Proton. Use of pre-made sequencing reagents avoids failures during initialization. On board Ion Torrent suite software does sequencing analysis at faster rate as compared with Ion PGM and Proton (S1 Table).

### Sequencing of multiple libraries from different panel sizes

Libraries from the same patients were prepared using different ampliseq panels (CHPv2, OCP and CCP) and run together on the S530 chip and independently on the S540 chip. Five runs were performed on the S530 chip consisting of different panels run together. On the S530 chip a mean depth of 217X and 1462X was achieved for the CCP and OCP panels, respectively, whereas mean depths of 150x, 209x and 4,762x were achieved for the CCP, OCP and CHPv2 when run together (S2 Table)

### Sequencing quality metrics for Ion S5XL

Using the Ion S5XL, an average sequencing output of 2 gigabases (Gb) and 9 Gb was obtained for 5 sequencing runs using the S530 and S540 chips, respectively. Averages of 29% of reads were polyclonal and 14% were of low quality on the S540 chip, whereas 34% of reads were polyclonal and 24% of reads were of low quality on the S530 chip and hence filtered. Therefore, on average in every sequencing run of 15 and 74 million reads on the S530 and S540 chips, respectively were of high quality (AQ20 or one error in 100 base pairs (bp) and provided useful sequence information). Comparatively, routine sequencing output observed by us for Ion PGM for the CHPv2 panel was 4 million reads, and for Ion Proton using a Ion PI HiQ sequencing chip for CCP or OCP panels was 9 Gb with 86 million reads. Average sequencing depth achieved for different panels using the S5XL were 450X (CCP), 1,500X (OCP) and 4,500X (CHPv2) (S2 Table).

### Data analysis on S5XL using Ion reporter and concordance with Ion PGM and Ion Proton

**Fig 2:**
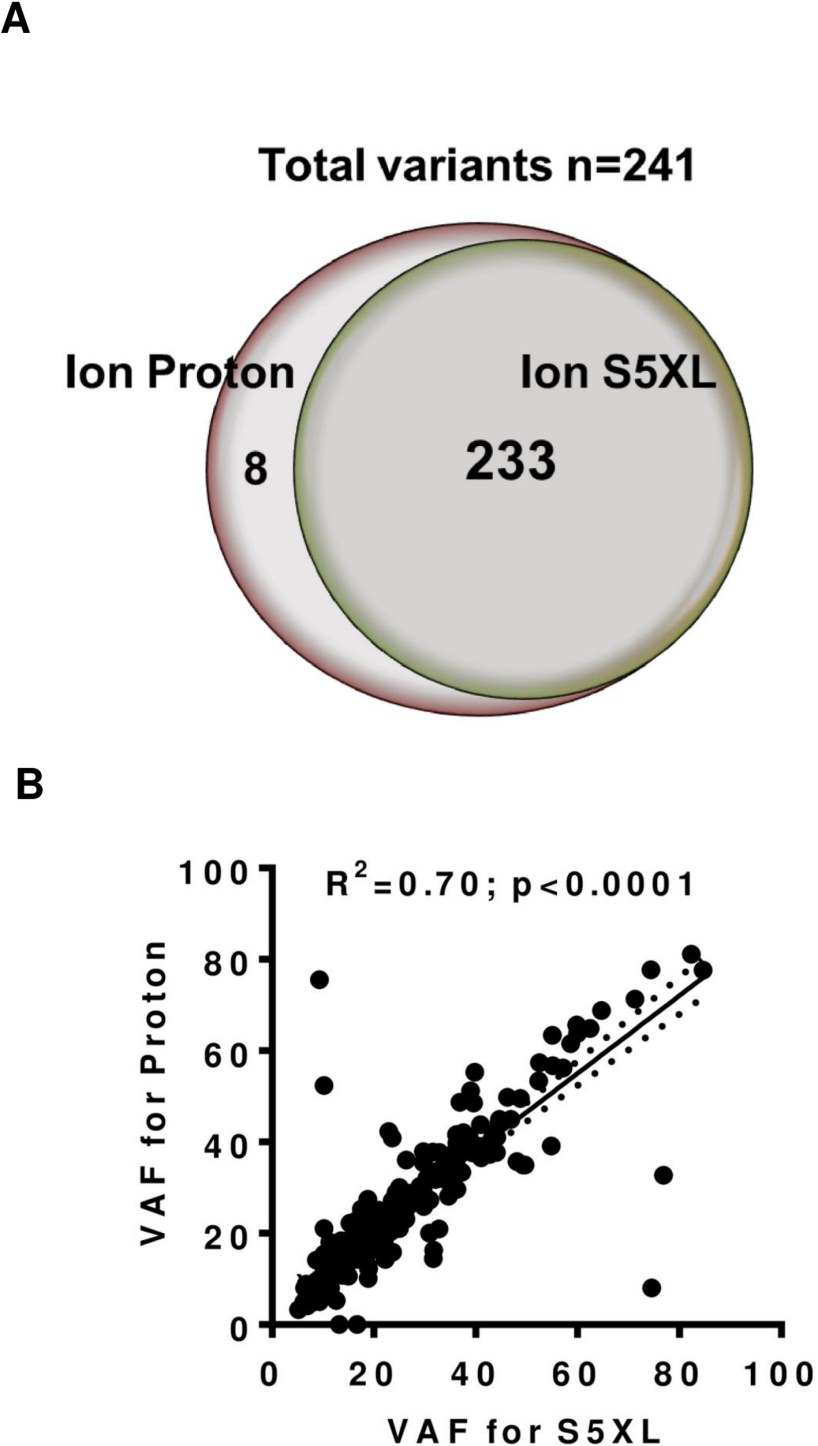
Variant call comparison between Ion Proton and Ion S5XL. (A) Venn diagram showed variant call comparison between Ion Proton and Ion S5Xl Ion S5XL showed 97% concordance for mutation detection with Ion Proton. Eight variant calls were missed by Ion S5XL due to low coverage in the variant region of the CCP panel compared to Ion Proton (B) Correlation between Variant allelic Fraction (VAF) of mutations detected on protons and Ion S5 XL (R^2^ = 0.70; p<0.0001) and each point on the graph represents a single variant analyzed by the Ion Proton and S5XL platforms.

A total of 241 variants (235 single nucleotide variants and 6 indels) expected in the studied cohort were compared between Ion PGM, Proton and S5XL (Fig 2A and S3 Table). The variant caller software efficiently detected somatic variants and indels present in samples tested on the Ion S5XL and showed 100% concordance with Ion PGM and 97% concordance with Ion Proton (R^2^ = 0.70; p<0.0001) for mutation detection, with only slight variation in allelic frequencies (Fig 2B and Table 1). Eight discordant calls (*RET*, *AMER1*, *GRM8*, *KIT*, *KRAS*, *MLL*, *MLL2*, *MLL3*) were missed by Ion S5XL due to low coverage in the variant region of the CCP panel compared to Ion Proton. All somatic mutations and indels called by Torrent suite onboard software on Ion S5XL for sequenced libraries were compared with Ion PGM and Protons for allelic frequency and coverage (S3 Table). IGV view of somatic mutations (*KRASp*.*G12V*), insertion (*SMAD4 p*. *L529_H530insR*) and deletion (*VHL del p*.H125fs*34) detected using three different panels on Ion S5XL is shown in S1 Fig. Variants detected using different panels on three different Ion Torrent instruments were compared (Table 1)

**Table 1:** Concordance of Ion S5XL with Ion PGM and Ion Proton for variants detected using different Ampliseq panels (CCP; OCP and CHPv2).

Variants	Platforms	Panel	Expected	Detected by Ion S5XL	% Concordance
SNVs	Ion PGM	CHPv2	26	26	100
	Ion Proton	Oncomine	65	65	100
		CCP	241	233	97
Indels	Ion Proton	CCP	6	6	100
Total			241	233	97

### Limit of detection study

**Fig 3:**
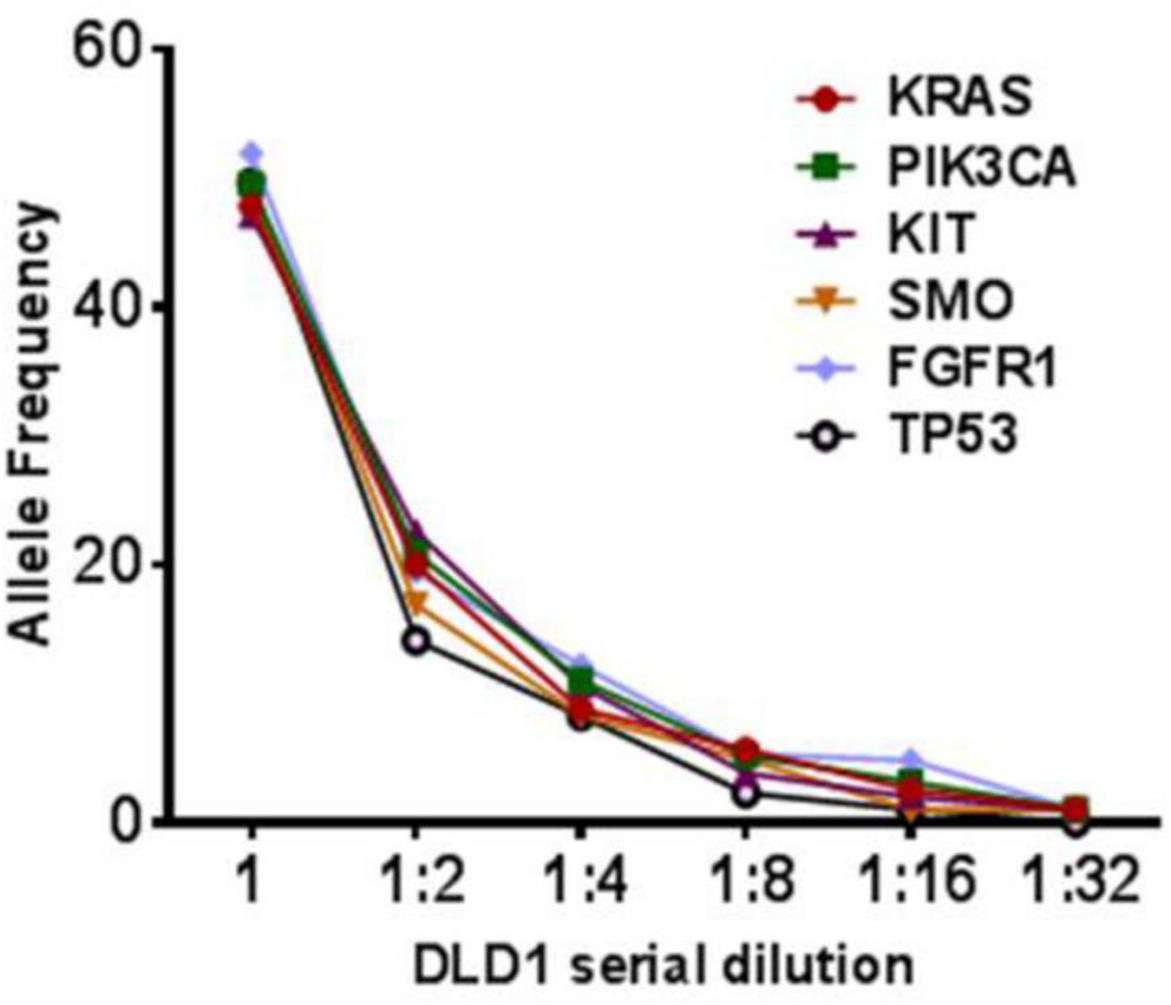
Limit of detection study performed on Ion S5 XL platform using S530 chip. Sensitivity study using DLD1 cell line serially diluted in wild type HL60 cell line DNA at 50, 25, 12.5, 6.25, 3.12, 1.50, and 0.75%. DLD1 cell line harbors heterozygous mutations in six different genes: *PIK3CA*, *KRAS*, *KIT*, *TP53*, *FGFR1* and *SMO*.

Libraries prepared using the CCP panels were used to determine the limit of detection of mutations on the Ion S5XL platform. DLD1 cell line FFPE DNA was sequentially diluted into H460 cell line DNA. The DLD1 cell line harbors heterozygous mutations in six different genes: *PIK3CA*, *KRAS*, *KIT*, *TP53*, *FGFR1* and *SMO*. All the expected mutations were detected in both runs at 50%, 25%, 10%, and 5%; *TP53* mutation was missed at 3.12% dilution (Fig 3 and S4 Table). This mutation was evident in the sequencing reads; however, owing to low sequencing depth they were not called by the variant caller software (TS v4.6.2). Therefore, a lower limit detection of 5% allelic frequency was established for the S5XL platform.

### Inter-run reproducibility

**Fig 4:**
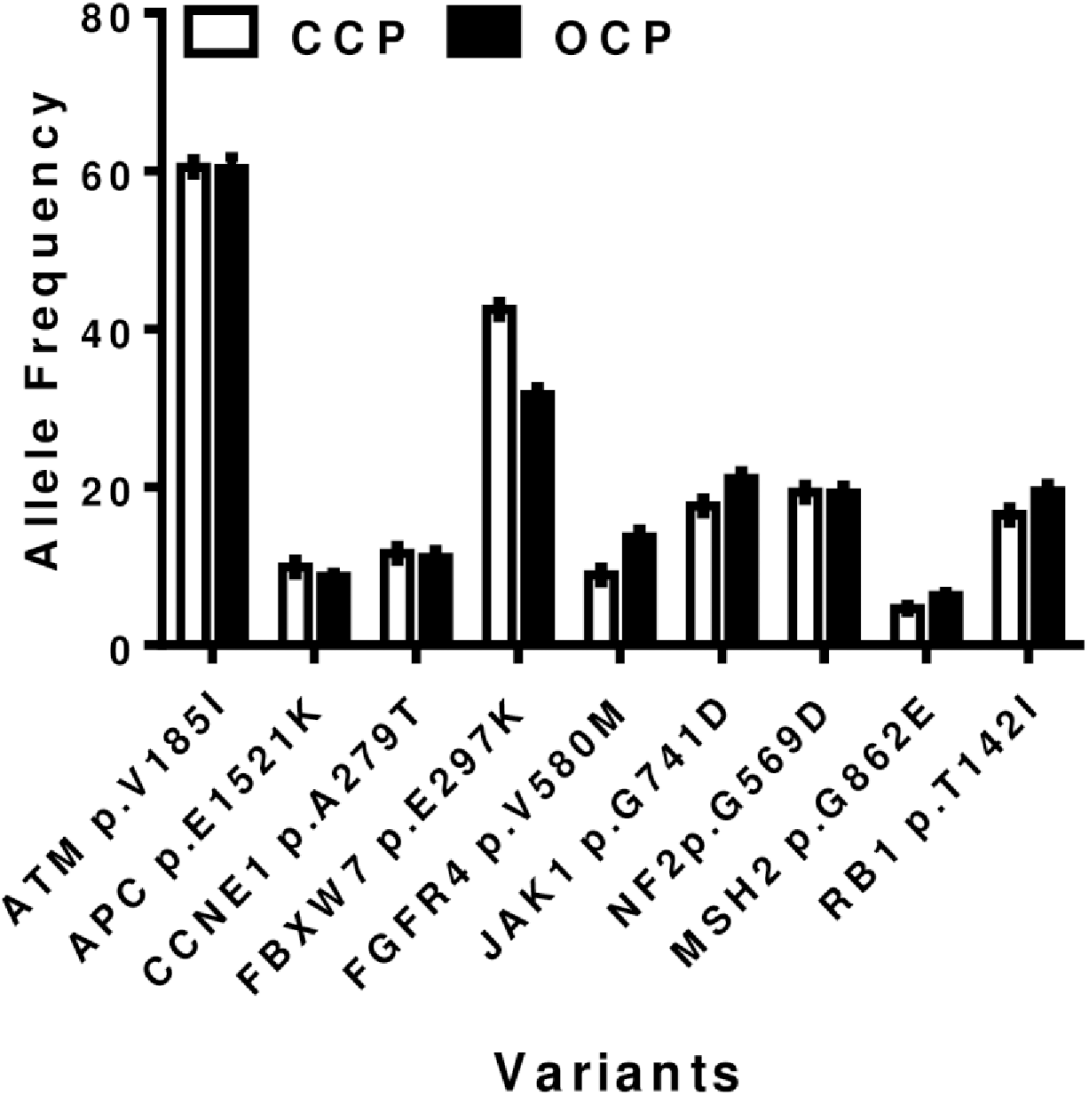
Inter-reproducibility studies performed on Ion S5 XL platform using S530 chip. Reproducibility studies using one normal tumor paired CCP libraries and one OCP libraries across two different sequencing runs on S5XL platform.

Inter-run reproducibility was determined by sequencing two normal tumor paired CCP libraries and two OCP libraries across two different sequencing runs on a S530 chip. A total of 9 variant hotspots present in both the OCP and CCP were compared. The results showed that each expected mutation was detected in every run with only slight variations in the allelic fractions (Fig 4 and S5 Table).

### Scalability and time study

Time and cost were compared at different steps from library preparation to sequencing for all three platforms tested. Libraries prepared from different panels were utilized on three different Ion torrent sequencers. Ion one touch 2 and Ion OneTouch ES were utilized for template preparation for sequencing on the Ion PGM and Ion Proton whereas the Ion Chef was used for the Ion S5XL. The difference in technologist time using OT2 (382 min) and Ion chef (282 min) is about 100 minutes per sample. In addition, template Ion Sphere for one chip using one touch OT2, whereas emulsion PCR for two sequencing runs and subsequent loading of 2 chips can be performed simultaneously with Ion Chef, drastically improving efficiency and decreasing the hands-on time requirement. Comparing sequencing capability, the Ion S5XL instrument is scalable to run different chips: S520 (output of 11 million reads), S530 (output of 20 million reads) and S540 (output of 80 million reads). The Ion PGM and Proton sequencers require preparation of initializing reagents and initialization of instruments which takes about 1 hour (h). In contrast, the Ion S5XL initialization is done using pre-made reagents shortening the initialization time to 40 min. Sequencing run time for 500 flows on Ion PGM and Ion Proton is 4:30 h, which can be shortened using HiQ reagents to 2:30 h for the Ion Proton whereas the Ion S5XL sequencing time per run is 2:30 h. Data analysis time for the Ion S5XL is faster 1 h (S520), 2.5 h (S530) and 5 h (S540) chip, respectively as compared to the Ion PGM which takes 3.5–5 hr (318 chip), and 8hr for Ion Proton (Ion PI chip), (S1 Fig and S1 Table).

## Discussion

Next generation sequencing based testing has made a major contribution to the characterization of somatic mutations in cancer genomes and the information derived from such analysis help guiding diagnosis and selection of therapy [3, 20–22]. Targeted NGS gene panels by allowing multiple targets to be analyzed simultaneously, often eliminate the need for reflex testing and preserve precious specimens by limiting the sample requirements for mutation analysis [4, 12]. A major limitation of current semiconductor sequencing-based NGS workflow is the laborious and time-consuming preparation of enriched DNA fragment or amplicon libraries for clonal isolation and amplification on beads [9(Gray, 2015 #5, 20, 23, 24]. In this study, we compared the performance of three semiconductor sequencing-based Ion Torrent platforms: Ion PGM, Ion Proton and Ion S5XL for detecting mutations in FFPE-derived tumor DNA. We also compared the analytical sensitivity, specificity, and capacity, ease of use and turnaround time. We show that the Ion S5XL sequencer provides simplified workflow, flexibility and scalability with a shorter turnaround time as compared with the other Ion Torrent platforms.

Earlier studies have shown that the targeted panels provide sufficient depth of coverage to detect minor allelic frequencies in a cost-effective manner, as compared with DNA whole genome or whole exome sequencing [4, 12, 18, 19, 25]. Multiplexing of samples on a sequencing runs is based on the size of the genomic area being screened by the panel and the sequencing capacity of the chip utilized in order to achieve the optimal sequencing depth necessary for confident variant detection and calling. In our workflow, we included sequencing of paired normal tissue or genomic DNA from peripheral blood to identify and filter the germline polymorphisms and identify somatic driver mutations more accurately. In addition, our filtering and annotating software interfaced with somatic and variant databases like COSMIC [26], dbSNP [27] and ClinVar [28] knowledgebase which helps to filter and annotate somatic and germline mutations [18, 29]. Thresholds for negative call (minimum 250x coverage) and positive calls (variant coverage: >25x, variant allelic frequency: >5%) were set on the basis of limit of detection studies conducted using serially diluted cell line on different platforms and panels used here. The set cut off helps to identify true somatic variants and avoid any false positives that can be generated at low allelic fraction (<5%) due to sequencing artifacts induced by the polymerase during PCR amplification and sequencing. Formalin fixation results in cytosine deamination in FFPE samples. The artifacts related to C>T/G>A changes were more frequently observed in low allele frequency variants (range <5%) and are tracked in our internal database for their occurrence across our entire sample cohort. Our set threshold of minimum 25X variant coverage and <5% VAF helps in minimizing these low level artifacts from being called. Treatment of DNA with UNG (Uracil N-glucosylase) has been proposed in the literature as a method to remove these artifacts [30]. Presence of homopolymer areas with same nucleotide is source of false-positive mutations during semiconductor sequencing due to lack of correlation of the extent of pH change to the nucleotides incorporated. Release of updated versions of Torrent suite software 5.0, set up thresholds for sequencing quality, sequencing depth, VAF, correlation with tumor percentage, strand bias and direct visualization of the sequence reads in Integrated Genome Viewer (IGV), will considerably help to decrease the rate of false-positive calls at these regions.

We ran different ampliseq panels individually (S540) and together on the same S5 chips (S530) on the Ion S5XL sequencer. The comparison between the three sequencing platforms was performed using 18 samples in which 235 somatic mutations and 6 indels had been previously identified using the Ion PGM and Ion Proton. All panels sequenced using Ion S5XL showed 100% and 97% concordance for mutation detection with Ion PGM and Ion Proton, respectively. The Torrent suite analysis software on-board S5XL sequencer detected all somatic variants and indels detected previously in an efficient manner with only slight variation in allelic fractions.

Implementation of a new NGS-based clinical assay requires high sensitivity, accuracy, precision, and specificity, in addition to high clinical utility [14, 18, 19, 21, 25, 31]. The 5% limit of detection for mutation analysis obtained with Ion S5XL was comparable to other platforms tested. High levels of reproducibility for mutation detection were demonstrated for the Ion S5XL using patient samples across two different sequencing runs. Low DNA input, cost and turnaround time are some of the critical factors that influence the choice of a NGS platform for a clinical diagnostic service. The Ion S5XL instrument supports three different types of chip with different output capacity allowing one to run smaller and larger panels on the same instrument; this feature offers flexibility to vary the number of samples per run without affecting cost. This choice of sequencing chips is very helpful to scale the number of samples and output according to the laboratory needs, which is a limitation with other Ion sequencers, specifically the Ion Proton. Use of the Ion Chef for the Ion S5XL automates template preparation, enrichment of ion spheres and chip loading, and therefore reduces the technologist time and chances of manual errors, without affecting the overall sequencing quality metrics and data. With simple cartridge-loaded reagents and a straightforward user interface, the Ion S5XL system makes NGS testing faster and more convenient. Premade sequencing reagents reduce the initialization time for the S5XL sequencer with <15 minutes of sequencer hands on time as compared to the Ion PGM and Proton. On board Torrent suite software on the Ion S5XL sequencer reduces the analysis time by half.

In conclusion, the Ion S5XL system simplifies the Ion torrent workflow and allows the use of different types of chips thereby facilitating the running of smaller and larger panels on the same instrument. The Ion S5XL also provides a shorter turnaround time and showed good concordance for variant calls with similar sensitivity and reproducibility to the Ion PGM and Proton platforms.

## Supporting information

S1 Fig IGV view of SNV and Indels detected by Ion S5XL. Variant call (somatic mutations) *KRAS* p.G12V, indels (Insertion SMAD4 p. L529_H530insR and deletion VHL p.H125fs*34) detected by Torrent Suite 4.6 (Ion S5XL from libraries prepared from same sample using three different Ampliseq panels (CHPv2, OCP and CCP). AF: % allelic fraction, Cov: coverage of variant call.
(TIF)

S1 Table Comparison of different parameters for Ion PGM, Ion Proton and Ion S5XL sequencing Platforms. (DOCX)

S2 Table Mapped reads/sample and mean depth for libraries prepared using different Ampliseq panel pooled together on S530 chip or independently on S540 chip and sequenced per run. (DOCX)

S3 Table Comparison of variant allelic fraction and coverage for samples ran on S5XL and Ion Proton using CCP, OCP and CHPV2 panel; NP: ccp panel not ran Ion S5XL; NC: Amplicons not covered in panel. (DOCX)

S4 Table Sensitivity study on Ion S5XL platform using serially diluted DLD 1 cell line positive for mutation in *PIK3CA, KRAS, KIT, TP53, FGFR1* and *SMO*. (DOCX)

S5 Table Inter reproducibility study using 1 CCP and 1 OCP normal tumor paired libraries from same patient. (DOCX)
