# Supplementary figures and images for: Versatile ion S5XL sequencer for targeted next generation sequencing of solid tumors in a clinical laboratory
Source: PLoS One. 2017 Aug 2;12(8):e0181968. doi: 10.1371/journal.pone.0181968 (PMC5540534; doi:10.1371/journal.pone.0181968)

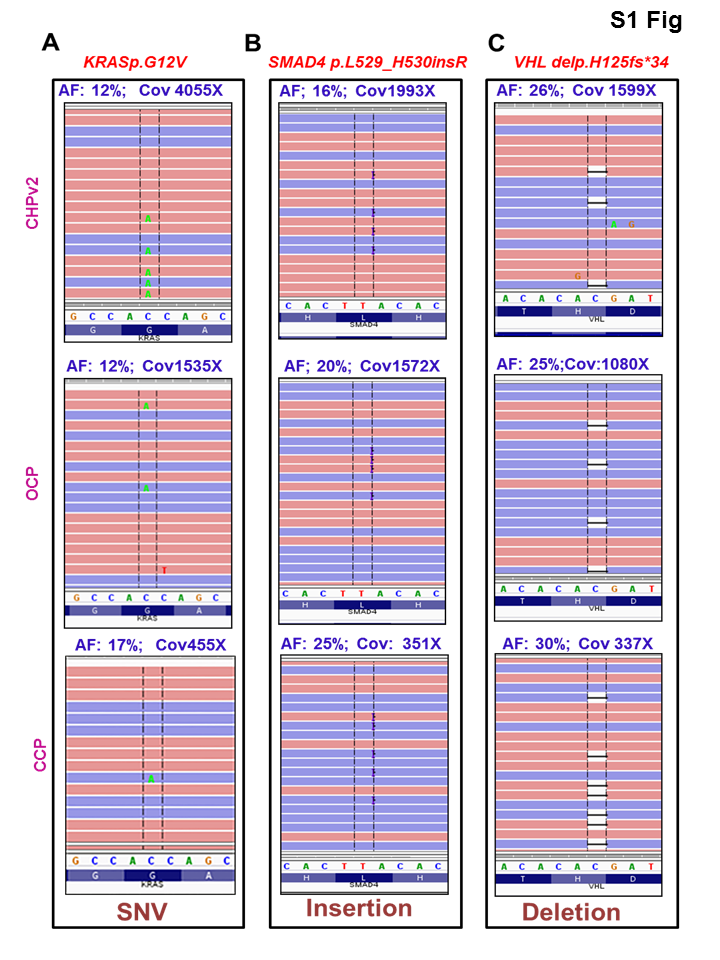

Supplement: S1 Fig — Variant call (somatic mutations) KRAS p.G12V, indels (Insertion SMAD4 p. L529_H530insR and deletion VHL p.H125fs*34) detected by Torrent Suite 4.6 (Ion S5XL from libraries prepared from same sample using three different Ampliseq panels (CHPv2, OCP and CCP). AF: % allelic fraction, Cov: coverage of variant call. (TIF) [file pone.0181968.s001.TIF]
