# Supplementary material for: Versatile ion S5XL sequencer for targeted next generation sequencing of solid tumors in a clinical laboratory
Source: PLoS One. 2017 Aug 2;12(8):e0181968. doi: 10.1371/journal.pone.0181968 (PMC5540534; doi:10.1371/journal.pone.0181968)
Supplement: S1 Table — (DOCX) [file pone.0181968.s002.docx]

| **Parameter** | **PGM** | **Proton** | **S5XL** |
| --- | --- | --- | --- |
| **Chip** | 314- 30-50Mb | Ion PI Chip v3-10Gb | S520 - 0.6-1Gb |
|  | 316- 300-500Mb |  | S530 - 3-4Gb |
|  | 318- 600-1Mb |  | S540 -10-15Gb |
| **Initialization reagents** | Need to be prepared | Need to be prepared | Pre made cartridge |
| **Initialization Time** | 60 min | 60 min | 40 min |
| **Instrument maintenance** | Daily/Weekly | Daily/Weekly | Not required |
| **Cleaning** | Chlorite/Water | Chlorite/Water | Self-cleaning after each run |
| **Nitrogen Tank** | Essential | Essential | Don’t needed |
| **Sequencing run time (500 flows)** | 4:30hr | 4:30hr | 2:30hr |
| **Template prep Procedure** | Manual; Ion One Touch OT2 | Manual; Ion One Touch OT2 | Automated; Ion Chef |
| **Ion spheres Enrichment** | Manual; Ion One Touch ES | Manual; Ion One Touch ES | Automated; Ion Chef |
| **Chip Loading** | Manual | Manual | Automated; Ion Chef |
| **Initial Sequencing QC** | 30 min | 30min | 20-30min |
| **Analysis time** | 314-2.5hr | 8hr | S520 - 1hr |
|  | 316 -3.5hr |  | S530 - 2.5hr |
|  | 318 – 5hr |  | S540 – 5hr |
| **Scalability/Flexibility for panels** | Small panels | Larger panels (OCP)/CCP | Flexible to run smaller and larger panels together on same chip or in independent runs |

**S1 Table:** Comparison of different parameters for Ion PGM, Ion Proton and Ion S5XL sequencing Platforms
