# Supplementary material for: Versatile ion S5XL sequencer for targeted next generation sequencing of solid tumors in a clinical laboratory
Source: PLoS One. 2017 Aug 2;12(8):e0181968. doi: 10.1371/journal.pone.0181968 (PMC5540534; doi:10.1371/journal.pone.0181968)
Supplement: S2 Table — (DOCX) [file pone.0181968.s003.docx]

| **S530** | | | **S540** | | |
| --- | --- | --- | --- | --- | --- |
| **Panels** | **Mapped reads/sample** | **Mean depth** | **Panels** | **Mapped reads/sample** | **Mean depth** |
| CCP-1(T+N)  OCP-2 (T) | CCP- 3,333,645  OCP- 3,593,608 | CCP-217x  OCP-1462x | CCP-10 (T+N) | 7,269,211 | 463.05x |
| OCP-4 (T) | 2,588,783 | 1037x | OCP-10 (T+N) | 3,683,881 | 1,515x |
| CCP-1 (T+N)  OCP- 1 (T) CHPV2- 10(T) | CCP- 1,433,172  OCP- 510,832  CHPV2-1,151,298 | CCP-150x  OCP-209x  CHPV2-4762x | CHPV2-10 (T+N)+ filler libraries prepared using OCP panel-20 | 978,256 | 4000x |

**S2 Table :** Mapped reads/sample and Mean depth for libraries prepared using different Ampliseq panel pooled together on S530 chip or independently on S540 chip and sequenced per run.

CCP: Comprehensive cancer panel, OCP: Oncomine panel, CHPV2: Cancer hotspot panel V2. T: Tumor, N: Normal
