# Supplementary material for: Versatile ion S5XL sequencer for targeted next generation sequencing of solid tumors in a clinical laboratory
Source: PLoS One. 2017 Aug 2;12(8):e0181968. doi: 10.1371/journal.pone.0181968 (PMC5540534; doi:10.1371/journal.pone.0181968)
Supplement: S3 Table — (DOCX) [file pone.0181968.s004.docx]

| **Sample**  **S3 Table:** Comparison of variant allelic fraction and coverage for samples ran on S5XL and Ion Proton using CCP, OCP and CHPV2 panel; NP: Not performed on Ion S5XL; NC: Amplicons not covered in panel | **Gene** | **HGVS** | **Type** | **Proton AF** | **Coverage** | **Ion S5XL CCP**  **AF (IR)** | **Coverage** | **Ion S5Xl**  **OCP AF (IR)** | **Coverage** | **Ion S5XL**  **CHPV2 AF (IR)** | **Coverage** |
| --- | --- | --- | --- | --- | --- | --- | --- | --- | --- | --- | --- |
| 1 | KRAS | G12V | SNV | 11.79 | 458 | 16.8 | 445 | 11.9 | 1535 | 11.86 | 1981 |
|  | TP53 | c.375+1G>A | SNV | 28.09 | 388 | 27.8 | 424 | 28.1 | 802 | 27.2 | 1987 |
|  | ESR1 | T311M | SNV | 22.07 | 1346 | 20.9 | 374 | NC | - | NC | - |
|  | EML4 | L49F | SNV | 20.8 | 1827 | 22.3 | 1251 | NC | - | NC | - |
|  | CSMD3 | T867A | SNV | 44.56 | 469 | 44.44 | 434 | NC | - | NC | - |
|  | CSMD3 | I279M | SNV | 7.06 | 821 | 7.69 | 819 | NC | - | NC | - |
|  | PKHD1 | R1422W | SNV | 22.53 | 537 | 21.6 | 375 | NC | - | NC | - |
|  |  |  |  |  |  |  |  |  |  |  |  |
| **2** | PTEN | L194* | SNV | 23.55 | 705 | 26.3 | 475 | 23.7 | 2000 | NC |  |
|  | VHL | del p.H125fs*34 | Indel | 29.64 | 587 | 29.6 | 337 | 25 | 1080 | 26.45 | 1599 |
|  | TET2 |  | Indel | 25.03 | 739 | 21.08 | 498 | 23.3 | 1016 | NC |  |
|  |  |  |  |  |  |  |  |  |  |  |  |
| **3** | KRAS | G13D | SNV | 15.56 | 482 | 16.3 | 595 | 15.4 | 1992 | 11.27 | 1987 |
|  | BAI3 | Q404K | SNV | 11.06 | 633 | 10.7 | 802 | NC | - | NC | - |
|  | LRP1B | V2139G | SNV | 8.16 | 674 | 8.52 | 869 | NC | - | NC | - |
|  | GNAS | GNAS | SNV | 6.71 | 38 | 10 | 650 | NC | - | NC | - |
|  | MDM2 | MDM2 | SNV | 6.34 | 22 | 7 | 531 | NC | - | NC | - |
|  |  |  |  |  |  |  |  |  |  |  |  |
| **4** | KIT | V532I | SNV | 13.17 | 243 | 0 | 118 | 0 | 1293 | 0.2 | 7992 |
|  | KRAS | Q61H | SNV | 16.71 | 377 | 0 | 399 | 0 | 1840 | 0.19 | 9120 |
|  | POT1 | Y223H | SNV | 23.64 | 588 | 15.9 | 289 | NC | - | NC | - |
|  |  |  |  |  |  |  |  |  |  |  |  |
| **5** | DCC | S383R | SNV | 6.39 | 954 | 8 | 453 | NC | - | NC | - |
|  | PIK3CA | E545K | SNV | 11.48 | 627 | 8 | 272 | 10.17 | 1986 | 9.88 | 1974 |
|  | SMAD4 | L529_H530insR | Indel | 20.99 | 1067 | 25 | 351 | 19.59 | 1572 | 15.86 | 1993 |
|  | KRAS | G12R | SNV | 9.29 | 226 | 5 | 246 | 12.8 | 1928 | 8.91 | 1997 |
|  |  |  |  |  |  |  |  |  |  |  |  |
| **6** | BRAF | V600E | SNV | 62.45 | 972 | 64.9 | 527 | 57.9 | 1987 | 64.21 | 1995 |
|  | PTEN | P95S | SNV | 82.33 | 651 | 81.1 | 291 | 83.5 | 589 | NC | - |
|  | MLL | S775F | SNV | 39.83 | 600 | 55.27 | 465 | NC | - | NC | - |
|  | ROS1 | H741Y | SNV | 84.62 | 143 | 77.6 | 321 | NC | - | NC | - |
|  | CSMD3 | F1801C | SNV | 23.44 | 1928 | 24.6 | 1141 | NC | - | NC | - |
|  | CSMD3 | R1765C | SNV | 71.26 | 2596 | 71.3 | 1255 | NC | - | NC | - |
|  | CSMD3 | R437K | SNV | 23.89 | 854 | 25.3 | 544 | NC | - | NC | - |
|  | EP400 | P518S | SNV | 48.1 | 158 | 35.7 | 475 | NC | - | NC | - |
|  | LRP1B | G4385E | SNV | 49.3 | 426 | 34.96 | 226 | NC | - | NC | - |
|  | LRP1B | G4385R | SNV | 49.65 | 429 | 34.96 | 226 | NC | - | NC | - |
|  | RNF213 | S3295F | SNV | 54.81 | 104 | 39.1 | 361 | NC | - | NC | - |
|  | SETD2 | V1431G | SNV | 16.67 | 228 | 22.9 | 445 | NC | - | NC | - |
|  | UGT1A1 | S306F | SNV | 44.67 | 1229 | 44.99 | 549 | NC | - | NC | - |
|  |  |  |  |  |  |  |  |  |  |  |  |
| **7** | CCNE1 | R85Q | SNV | 28.97 | 887 | 27.3 | 1348 | NC | - | NC | - |
|  | ERG | R285H | SNV | 13.99 | 922 | 13.53 | 813 | NC | - | NC | - |
|  | KRAS | G12V | SNV | 22.06 | 485 | 18.68 | 514 | 22.09 | 1978 | 22.17 | 1980 |
|  | PTPRT | V846I | SNV | 13.14 | 601 | 15.34 | 769 | NC | - | NC | - |
|  | TP53 | R248L | SNV | 25.4 | 559 | 24.82 | 854 | 25.47 | 1716 | 28.7 | 1946 |
|  |  |  |  |  |  |  |  |  |  |  |  |
| **8** | BRAF | N486D | SNV | 8.27 | 786 | 8.43 | 581 | NC | - | NC | - |
|  | KRAS | G12V | SNV | 44.17 | 566 | 41.02 | 334 | 41.47 | 1987 | 43.09 | 1975 |
|  | TP53 | R249S | SNV | 20.37 | 766 | 23.36 | 655 | 16.87 | 1986 | 18.47 | 1998 |
|  | MLL3 | A3556S | SNV | 22.27 | 238 | 14.29 | 426 | NC | - | NC | - |
|  | ABL2 | V571L | SNV | 18.11 | 867 | 18.27 | 761 | NC | - | NC | - |
|  | ITGB3 | R119L | SNV | 19.44 | 252 | 16.8 | 518 | NC | - | NC | - |
|  |  |  |  |  |  |  |  |  |  |  |  |
| **9** | AFF3 | P781S | SNV | 33.67 | 796 | 34.6 | 779 | NC | - | NC | - |
|  | APC | N1958S | SNV | 15.41 | 279 | 18.78 | 281 | 23.1 | 1997 | NC | - |
|  | ATR | I2345T | SNV | 34.65 | 127 | 28.1 | 264 | NC | - | NC | - |
|  | BCL9 | .M1277T | SNV | 29.7 | 202 | 37.9 | 408 | NC | - | NC | - |
|  | BRAF | P676S | SNV | 11.68 | 736 | 13.9 | 603 | NC | - | NC | - |
|  | BRAF | S337L | SNV | 25.95 | 1137 | 23.3 | 1360 | NC | - | NC | - |
|  | CASC5 | H920L | SNV | 24.67 | 523 | 24.03 | 362 | NC | - | NC | - |
|  | CDH20 | G168R | SNV | 35.55 | 1249 | 32.5 | 1209 | NC | - | NC | - |
|  | CREBBP | S671L | SNV | 32.07 | 605 | 31.82 | 330 | NC | - | NC | - |
|  | CSF1R | G669D | SNV | 16.91 | 343 | 15.2 | 487 | NC | - | NC | - |
|  | CSF1R | W408* | SNV | 26.29 | 194 | 23.1 | 268 | 20.4 | 914 | NC | - |
|  | CSMD3 | P3320S | SNV | 33.69 | 748 | 33.3 | 880 | NC | - | NC | - |
|  | CSMD3 | R2688C | SNV | 18.92 | 867 | 12.2 | 628 | NC | - | NC | - |
|  | CYP2C19 | F428L | SNV | 39.37 | 508 | 37.6 | 279 | NC | - | NC | - |
|  | DCC | T1105I | SNV | 35.66 | 959 | 34.5 | 1012 | NC | - | NC | - |
|  | DICER1 | P623S | SNV | 18.45 | 1577 | 20 | 1622 | NC | - | NC | - |
|  | EP300 | R568W | SNV | 16.21 | 1351 | 14.7 | 871 | NC | - | NC | - |
|  | ERBB4 | G1217E | SNV | 32.26 | 1302 | 33.5 | 1195 | NC | - | NC | - |
|  | ERBB4 | G870R | SNV | 36.25 | 618 | 29.6 | 459 | NC | - | NC | - |
|  | FANCG | R613W | SNV | 55.1 | 1058 | 56.7 | 1292 | NC | - | NC | - |
|  | FGFR1 | E358K | SNV | 18.68 | 1001 | 20.05 | 1262 | 20.31 | 1999 | NC | - |
|  | IL7R | G424E | SNV | 36.15 | 1087 | 35.12 | 1512 | NC | - | NC | - |
|  | ITGB2 | R586Q | SNV | 36 | 175 | 36.7 | 846 | NC | - | NC | - |
|  | LIFR | G835E | SNV | 35.28 | 1060 | 33.2 | 799 | NC | - | NC | - |
|  | LIFR | E129K | SNV | 31.32 | 463 | 32.9 | 334 | NC | - | NC | - |
|  | LPHN3 | D712N | SNV | 19.83 | 242 | 19.49 | 336 | NC | - | NC | - |
|  | LPHN3 | M762I | SNV | 44.04 | 545 | 37.7 | 355 | NC | - | NC | - |
|  | LPHN3 | L920F | SNV | 17.76 | 473 | 19.1 | 466 | NC | - | NC | - |
|  | LPP | P335L | SNV | 23.84 | 495 | 24.3 | 529 | NC | - | NC | - |
|  | LRP1B | M3934I | SNV | 29.34 | 1493 | 29.4 | 1028 | NC | - | NC | - |
|  | LRP1B | E2849D | SNV | 37.28 | 869 | 33.4 | 595 | NC | - | NC | - |
|  | MARK1 | M154I | SNV | 31 | 1629 | 27.3 | 1254 | NC | - | NC | - |
|  | MET | R1184Q | SNV | 18.98 | 411 | 23.77 | 408 | NC | - | NC | - |
|  | MSH2 | S233F | SNV | 30.05 | 1318 | 37.05 | 1228 | NC | - | NC | - |
|  | MYH11 | A805fs*9 | Indel | 86.54 | 52 | 42.3 | 356 | NC | - | NC | - |
|  | MYH11 | E1138K | SNV | 16.05 | 243 | 15.85 | 456 | NC | - | NC | - |
|  | NF1 | .L1300P | SNV | 42.81 | 640 | 37.32 | 477 | 43.35 | 1993 | NC | - |
|  | NF1 | R2349C | SNV | 20.48 | 1133 | 23.3 | 699 | 21.62 | 1411 | NC | - |
|  | NF1 | 1G>A | SNV | 20.74 | 516 | 18.1 | 315 | 19.92 | 1059 | NC | - |
|  | PALB2 | S319F | SNV | 46.26 | 990 | 49.8 | 793 | NC | - | NC | - |
|  | PAX5 | R31Q | SNV | 57.17 | 544 | 56.2 | 535 | NC | - | NC | - |
|  | PDE4DIP | D1624N | SNV | 14.89 | 1350 | 15.14 | 1189 | NC | - | NC | - |
|  | PDGFRB | T192A | SNV | 18.82 | 255 | 27.44 | 277 | NC | - | NC | - |
|  | PHOX2B | E130K | SNV | 36.23 | 69 | 41.59 | 630 | NC | - | NC | - |
|  | PKHD1 | S3787L | SNV | 38.68 | 866 | 41.22 | 621 | NC | - | NC | - |
|  | POT1 | K430* | SNV | 23.27 | 795 | 24.9 | 510 | NC | - | NC | - |
|  | PRDM1 | T275P | SNV | 21.73 | 382 | 19.62 | 372 | NC | - | NC | - |
|  | PTPRD | L1288F | SNV | 21.54 | 311 | 24.03 | 233 | NC | - | NC | - |
|  | PTPRT | G848R | SNV | 36.17 | 871 | 34.78 | 946 | NC | - | NC | - |
|  | PTPRT | G848E | SNV | 35.92 | 863 | 34.78 | 946 | NC | - | NC | - |
|  | RAD50 | S128C | SNV | 24.51 | 816 | 21.49 | 656 | NC | - | NC | - |
|  | RB1 | R556* | SNV | 31.58 | 228 | 37.8 | 290 | 51.03 | 872 | NC | - |
|  | RUNX1T1 | E407K | SNV | 20.44 | 274 | 22.6 | 260 | NC | - | NC | - |
|  | SAMD9 | R1333K | SNV | 15.95 | 1354 | 16.2 | 912 | NC | - | NC | - |
|  | SDHA | H363L | SNV | 36.19 | 536 | 38.5 | 978 | NC | - | NC | - |
|  | SMARCA4 | D1350N | SNV | 33.99 | 609 | 33.5 | 1591 | NC | - | NC | - |
|  | TET2 | R1404* | SNV | 37.59 | 689 | 42 | 452 | 41.1 | 1994 | NC | - |
|  | TLR4 | R606Q | SNV | 48.77 | 1015 | 49.5 | 930 | NC | - | NC | - |
|  | TNFRSF14 | I214V | SNV | 26.38 | 163 | 36 | 247 | NC | - | NC | - |
|  | TRRAP | E2532K | SNV | 13.9 | 784 | 14.8 | 2000 | NC | - | NC | - |
|  | ZNF521 | D732H | SNV | 29.04 | 1071 | 30.27 | 978 | NC | - | NC | - |
|  |  |  |  |  |  |  |  |  |  |  |  |
| **10** | EGFR | G>T | SNV | 15.01 | 393 | 14.01 | 150 | 18.64 | 242 | 58.67 | 467 |
|  | RET | L730I | SNV | 21.25 | 160 | 0 | 156 | NC | - | NC | - |
|  | TP53 | E221* | SNV | 25.17 | 302 | 25.8 | 262 | 28 | 921 | 24.14 | 522 |
|  | AKT3 | S370P | SNV | 13.73 | 306 | 15.11 | 239 | NC | - | NC | - |
|  | BCR | R813S | SNV | 30.92 | 207 | 20 | 280 | NC | - | NC | - |
|  | FLT1 | G886V | SNV | 17.32 | 231 | 18.9 | 280 | NC | - | NC | - |
|  | NF1 | G823* | SNV | 29.8 | 255 | 25.9 | 327 | 37 | 972 | NC | - |
|  | PDGFRB | P123H | SNV | 11.09 | 442 | 8.18 | 318 | NC | - | NC | - |
|  | EPHA3 | T84R | SNV | 11.31 | 168 | 18.08 | 277 | NC | - | NC | - |
|  | ADAMTS20 | I263T | SNV | 18.87 | 159 | 12.86 | 270 | NC | - | NC | - |
|  | AFF3 | D67Y | SNV | 16.73 | 281 | 14.29 | 305 | NC | - | NC | - |
|  | AKAP9 | E1971A | SNV | 11.32 | 212 | 14 | 313 | NC | - | NC | - |
|  | ARID1A | R1671P | SNV | 18.11 | 944 | 18.15 | 281 | NC | - | NC | - |
|  | ATRX | K2261R | SNV | 5.23 | 306 | 3.33 | 320 | NC | - | NC | - |
|  | BAI3 | P742T | SNV | 16.64 | 631 | 16.9 | 242 | NC | - | NC | - |
|  | BRIP1 | K577N | SNV | 15.32 | 333 | 22.22 | 250 | NC | - | NC | - |
|  | CD79B | A149V | SNV | 15.02 | 213 | 10.64 | 247 | NC | - | NC | - |
|  | CDH20 | D666N | SNV | 6.88 | 378 | 6.55 | 329 | NC | - | NC | - |
|  | CSMD3 | Q3439H | SNV | 10.2 | 343 | 21.05 | 257 | NC | - | NC | - |
|  | CSMD3 | G1768V | SNV | 8.72 | 860 | 14.12 | 270 | NC | - | NC | - |
|  | EPHB6 | A462S | SNV | 31.66 | 259 | 14.47 | 276 | NC | - | NC | - |
|  | ETV1 | E237* | SNV | 11.17 | 716 | 11.24 | 356 | NC | - | NC | - |
|  | AMER1 | D833V | SNV | 33.9 | 876 | 34.12 | 296 | NC | - | NC | - |
|  | AMER1 | G739R | SNV | 6.83 | 410 | 0 | 86 | NC | - | NC | - |
|  | GRM8 | S752* | SNV | 10.31 | 320 | 0 | 65 | NC | - | NC | - |
|  | GRM8 | Q679K | SNV | 29.73 | 185 | 35.45 | 210 | NC | - | NC | - |
|  | ICK | G112V | SNV | 30.38 | 655 | 32 | 288 | NC | - | NC | - |
|  | KAT6B | D896N | SNV | 14.7 | 483 | 18.04 | 294 | NC | - | NC | - |
|  | KEAP1 | R240L | SNV | 25.86 | 116 | 25 | 240 | NC | - | NC | - |
|  | LRP1B | Q409K | SNV | 26.93 | 1021 | 28.75 | 240 | NC | - | NC | - |
|  | LRP1B | R2609* | SNV | 10.64 | 1316 | 14.17 | 487 | NC | - | NC | - |
|  | PRDM1 | 2A>G | SNV | 10.5 | 524 | 11.29 | 186 | NC | - | NC | - |
|  | RNF213 | E746V | SNV | 15.14 | 535 | 18.06 | 272 | NC | - | NC | - |
|  | WAS | A134S | SNV | 18.59 | 199 | 21.82 | 210 | NC | - | NC | - |
|  |  |  |  |  |  |  |  |  |  |  |  |
| **11** | ATM | p.V185I | SNV | 58.63 | 1141 | 61.5 | 533 | 61.78 | 191 | CMS46 negative | 467 |
|  | GNAS | p.A249T | SNV | 36.43 | 479 | 41.03 | 290 | NC |  | NC |  |
|  | APC | p.P2740L | SNV | 19.43 | 880 | 21.82 | 417 | 14.62 | 260 | NC | 522 |
|  | APC | p.A2298T | SNV | 9.31 | 816 | 7.55 | 384 | 11.08 | 325 | NC | - |
|  | APC | p.E1521K | SNV | 6.7 | 638 | 8.88 | 383 | 8.22 | 998 | NC | - |
|  | APC | p.P1733S | SNV | 19.58 | 720 | 24.04 | 470 | 21.96 | 642 | NC | - |
|  | CCNE1 | p.A279T | SNV | 10.14 | 1252 | 12.6 | 500 | 12.08 | 265 | NC | - |
|  | CSF1R | p.P87L | SNV | 10.1 | 346 | 9.81 | 265 | NC | - | NC | - |
|  | EML4 | p.P199S | SNV | 19.9 | 312 | 19.8 | 271 | NC | - | NC | - |
|  | ERBB3 | p.K472R | SNV | 74.55 | 503 | 80 | 335 | NC | - | NC | - |
|  | ERBB4 | p.E1287K | SNV | 60 | 255 | 63.8 | 319 | NC | - | NC | - |
|  | FBXW7 | p.E297K | SNV | 37.5 | 996 | 41.5 | 418 | 32.7 | 213 | NC | - |
|  | FGFR4 | p.V580M | SNV | 10.98 | 246 | 7.93 | 227 | 12.7 | 257 | NC | - |
|  | IL7R | p.E47K | SNV | 10.4 | 1258 | 9.89 | 566 | NC | - | NC | - |
|  | JAK1 | p.G741D | SNV | 18.6 | 338 | 18.6 | 349 | 22.12 | 213 | NC | - |
|  | MLL1 | G>A; | splice | 22.5 | 561 | 0 | 25 | NC | - | NC | - |
|  | MLL2 | p.T4386I | SNV | 12.9 | 225 | 0 | 46 | NC | - | NC | - |
|  | MLL3 | p.A1619V | SNV | 12.6 | 1927 | 0 | 26 | NC | - | NC | - |
|  | NF2 | p.G569D | SNV | 13.59 | 552 | 18.4 | 276 | 18.2 | 257 | NC | - |
|  | NTRK3 | p.V780I | SNV | 6.87 | 393 | 5.58 | 233 | NC | - | NC | - |
|  | PDGFRB | P1026S | SNV | 8.58 | 1259 | 7.93 | 593 | NC | - | NC | - |
|  | PTPN11 | p.V243I | SNV | 29.52 | 210 | 29.8 | 278 | NC | - | NC | - |
|  | TOP1 | p.V684I | SNV | 13.69 | 928 | 12.4 | 387 | NC | - | NC | - |
|  | AFF3 | p.S273F | SNV | 22.39 | 335 | 22.8 | 285 | NC | - | NC | - |
|  | ASXL1 | p.D104N | SNV | 12.37 | 784 | 13.5 | 457 | NC | - | NC | - |
|  | ATF1 | p.G145D | SNV | 13.65 | 520 | 12.8 | 350 | NC | - | NC | - |
|  | ATRX | p.A1407T | SNV | 21.24 | 306 | 22.7 | 299 | NC | - | NC | - |
|  | BIRC3 | p.A471T | SNV | 20.92 | 650 | 20.66 | 334 | NC | - | NC | - |
|  | BLM | p.E929K | SNV | 17.03 | 411 | 21.51 | 251 | NC | - | NC | - |
|  | BRIP1 | p.G840R | SNV | 32.88 | 882 | 37.7 | 487 | NC | - | NC | - |
|  | CDH5 | p.A19T | SNV | 55.03 | 149 | 63.4 | 264 | NC | - | NC | - |
|  | CTNNA1 | p.K81R | SNV | 11.01 | 1890 | 8.34 | 755 | NC | - | NC | - |
|  | CYLD | del p.V165fs | indel | 12.61 | 571 | 5.29 | 289 | NC | - | NC | - |
|  | DICER1 | p.T236I | SNV | 11.07 | 551 | 10.7 | 269 | NC | - | NC | - |
|  | DST | p.E1730K | SNV | 17.21 | 366 | 16.86 | 266 | NC | - | NC | - |
|  | EPHA7 | p.P443S | SNV | 18.07 | 581 | 15.69 | 306 | NC | - | NC | - |
|  | ERCC5 | p.W950* | SNV | 14 | 607 | 16.8 | 250 | NC | - | NC | - |
|  | ERCC5 | p.P677L | SNV | 19.42 | 448 | 16.5 | 466 | NC | - | NC | - |
|  | ERCC5 | p.E1135K | SNV | 17.51 | 337 | 25.25 | 298 | NC | - | NC | - |
|  | ETV1 | p.S247N | SNV | 40.75 | 697 | 39.07 | 366 | NC | - | NC | - |
|  | FANCC | p.S184L | SNV | 11.14 | 341 | 13.01 | 246 | NC | - | NC | - |
|  | FANCG | p.Y249C | SNV | 13.86 | 505 | 10.77 | 390 | NC | - | NC | - |
|  | FOXP4 | G>A | splice | 23.77 | 122 | 25.66 | 212 | NC | - | NC | - |
|  | GATA3 | p.S373F | SNV | 35.71 | 140 | 34.88 | 229 | 38.7 | 284 | NC | - |
|  | KAT6A | p.P165S | SNV | 16.15 | 322 | 19.43 | 275 | NC | - | NC | - |
|  | KAT6A | p.A1741V | SNV | 13.85 | 231 | 13.13 | 199 | NC | - | NC | - |
|  | KDM6A | p.P1327S | SNV | 24.39 | 578 | 28.3 | 265 | NC | - | NC | - |
|  | LAMP1 | p.A265V | SNV | 32.79 | 122 | 20.93 | 215 | NC | - | NC | - |
|  | LPHN3 | p.A266V | SNV | 10.38 | 703 | 11.94 | 310 | NC | - | NC | - |
|  | MAML2 | p.G524E | SNV | 22.87 | 223 | 42.25 | 271 | NC | - | NC | - |
|  | MAP3K7 | p.A323V | SNV | 16.52 | 351 | 20.77 | 183 | NC | - | NC | - |
|  | MSH2 | p.G862E | SNV | 6.9 | 1344 | 4.15 | 458 | 5.78 | 273 | NC | - |
|  | MSH6 | p.G1157D | SNV | 59.8 | 234 | 65.6 | 233 | NC | - | NC | - |
|  | NSD1 | p.S1654F | SNV | 7.9 | 1288 | 4.88 | 594 | NC | - | NC | - |
|  | NUMA1 | p.G406D | SNV | 25 | 264 | 30.07 | 243 | NC | - | NC | - |
|  | NUMA1 | G>A | splice | 18.89 | 450 | 10.09 | 209 | NC | - | NC | - |
|  | NUP214 | p.S1271N | SNV | 29.29 | 297 | 26.52 | 281 | NC | - | NC | - |
|  | NUP214 | p.E266K | SNV | 6.21 | 435 | 4.95 | 202 | NC | - | NC | - |
|  | PBRM1 | G>A | splice | 8.01 | 737 | 7.41 | 270 | NC | - | NC | - |
|  | PBRM1 | p.P1118S | SNV | 15.54 | 624 | 14.03 | 278 | NC | - | NC | - |
|  | PKHD1 | p.S146N | SNV | 23.58 | 318 | 27.14 | 299 | NC | - | NC | - |
|  | PMS1 | p.P749S | SNV | 52.35 | 405 | 53.37 | 278 | NC | - | NC | - |
|  | PRDM1 | p.G788E | SNV | 20.24 | 242 | 16.47 | 285 | NC | - | NC | - |
|  | PRKAR1A | p.A111T | SNV | 14.18 | 402 | 14.15 | 325 | NC | - | NC | - |
|  | PTPRD | G>A | splice | 11.9 | 714 | 10.12 | 512 | NC | - | NC | - |
|  | PTPRT | p.L422F | SNV | 11.9 | 254 | 11.48 | 283 | NC | - | NC | - |
|  | RB1 | p.T142I | SNV | 10.23 | 665 | 15.48 | 268 | 18.52 | 135 | 12.89 | 1164 |
|  | REL | p.G161D | SNV | 16.9 | 420 | 22.79 | 236 | NC | - | NC | - |
|  | RNF213 | p.A694V | SNV | 28.72 | 94 | 28.35 | 227 | NC | - | NC | - |
|  | RNF213 | p.E1255K | SNV | 10.53 | 570 | 9.41 | 340 | NC | - | NC | - |
|  | RNF213 | p.S3511N | SNV | 10.21 | 333 | 52.35 | 279 | NC | - | NC | - |
|  | SYNE1 | p.E4032K | SNV | 26.51 | 498 | 25.61 | 264 | NC | - | NC | - |
|  | TAF1 | p.A1489T | SNV | 20.25 | 731 | 22.68 | 313 | NC | - | NC | - |
|  | TCF12 | p.A649V | SNV | 24.36 | 472 | 28.9 | 218 | NC | - | NC | - |
|  | TET1 | p.H2040Y | SNV | 14.45 | 353 | 10.79 | 239 | NC | - | NC | - |
|  | TRRAP | p.P2528L | SNV | 31.69 | 142 | 16.33 | 247 | NC | - | NC | - |
|  |  |  |  |  |  |  |  |  |  |  |  |
| **12** | IDH1 | p.R132H | SNV | 46.9 | 527 | 45 | 235 | 44.95 | 1507 | 44.5 | 1997 |
|  | PIK3CA | p.Y1021H | SNV | 9.6 | 385 | 7.95 | 261 | 6.25 | 1005 | 9.6 | 1998 |
|  |  |  |  |  |  |  |  |  |  |  |  |
| **13** | CTNNB1 | p.S37F | SNV | 34.1 | 446 | NP |  | 31.7 | 1572 | 30.2 | 1988 |
|  | KIT | p.N566D | SNV | 10.6 | 1533 |  |  | 12.05 | 2000 | 9.99 | 2000 |
|  | TP53 | p.Q331* | SNV | 64.7 | 935 |  |  | 68.8 | 1471 | NC | - |
|  | NF2 | p.R310I | SNV | 23.3 | 1195 |  |  | 20.35 | 1995 | NC | - |
|  | TSC1 | p.S427F | SNV | 37.3 | 472 |  |  | 41.61 | 1997 | NC | - |
|  | GATA3 | del p.S238fs | indel | 23.6 | 110 |  |  | 40.9 | 286 | NC | - |
|  |  |  |  |  |  |  |  |  |  |  |  |
| **14** | MAP2K1 | p.G128D | SNV | 18.6 | 156 | NP |  | 17 | 884 | NC | - |
|  |  |  |  |  |  |  |  |  |  |  |  |
| **15** | EGFR | L858R | SNV | 28.2 | N/A | NC |  | NP | - | 28.44 | 1990 |
|  | EGFR | E709K | SNV | 27.3 |  |  |  |  |  | 27.9 | 1991 |
|  |  |  |  |  |  |  |  |  |  |  |  |
| **16** | TP53 | R175H | SNV | 43 |  | NP |  | NP | - | 40.3 | 2000 |
|  |  |  |  |  |  |  |  |  |  |  |  |
| **17** | NF2 | p.Q333* | SNV | 74.4 | 242 | NP |  | 77.67 | 1012 | NC | - |
|  | MYCN | p.A306S | SNV | 15.1 | 491 |  |  | 14.74 | 1913 | NC | - |
|  | TP53 | R283C | SNV | 52.5 | 569 |  |  | 57.38 | 1999 | 52.3 | 1999 |
|  |  |  |  |  |  |  |  |  |  |  |  |
| **18** | TP53 | p.R273C | SNV | 41.1 | 712 | NP |  | 36.57 | 1206 | 40.2 | 1997 |
|  | TP53 | p.R282W | SNV | 36.9 | 710 |  |  | 48.68 | 1785 | 39.6 | 1795 |
|  | APC | p.V1804G | SNV | 76.9 | 52 |  |  | 32.72 | 654 | NC | - |
|  | BAP1 | p.R114H | SNV | 41.7 | 336 |  |  | 38.49 | 977 | NC | - |
|  | KDR | p.T77IM | SNV | 39 | 369 |  |  | 51.24 | 1454 | NC | - |
|  | NOTCH1 | p.G364D | SNV | 43.4 | 106 |  |  | 41.89 | 274 | NC | - |
|  | PPARG | p.T487M | SNV | 38.4 | 229 |  |  | 38.15 | 1198 | NC | - |
|  | PPARG | p.E352K | SNV | 36.3 | 317 |  |  | 39.77 | 1325 | NC | - |
|  | PTCH1 | p.G1212D | SNV | 40.9 | 330 |  |  | 43.75 | 280 | NC | - |
