# Supplementary material for: Versatile ion S5XL sequencer for targeted next generation sequencing of solid tumors in a clinical laboratory
Source: PLoS One. 2017 Aug 2;12(8):e0181968. doi: 10.1371/journal.pone.0181968 (PMC5540534; doi:10.1371/journal.pone.0181968)
Supplement: S4 Table — (DOCX) [file pone.0181968.s005.docx]

| **Dilution** | **Variant** | **Expected AF (%)** | **AF (%)** |
| --- | --- | --- | --- |
| Undiluted | *KRAS p.G13D* | 48 | 47.86 |
|  | *PIK3CA p.D549N* | 50 | 49.57 |
|  | *KIT p.V532I* | 47 | 47.16 |
|  | *SMO p.T640A* | 50 | 49.50 |
|  | *FGFR1p.A266S* | 52 | 51.94 |
|  | *TP53 p.S241F* | 50 | 49.67 |
|  |  |  |  |
| 50 % (1:1) | *KRAS p.G13D* | 24 | 20.00 |
|  | *PIK3CA p.D549N* | 25 | 20.90 |
|  | *KIT p.V532I* | 23.5 | 22.60 |
|  | *SMO p.T640A* | 25 | 16.90 |
|  | *FGFR1p. A266S* | 26 | 19.44 |
|  | *TP53 p.S241F* | 25 | 14.15 |
|  |  |  |  |
| 25 % (1:3) | *KRAS p.G13D* | 12 | 8.82 |
|  | *PIK3CA p.D549N* | 12.5 | 11.03 |
|  | *KIT p.V532I* | 11.7 | 10.61 |
|  | *SMO p.T640A* | 12.5 | 8.33 |
|  | *FGFR1p. A266S* | 13 | 12.22 |
|  | *TP53 p.S241F* | 12.5 | 8.27 |
|  |  |  |  |
| 12.5% (1:8) | *KRAS p.G13D* | 6 | 5.65 |
|  | *PIK3CA p.D549N* | 6.25 | 5.19 |
|  | *KIT p.V532I* | 5.87 | 3.86 |
|  | *SMO p.T640A* | 6.25 | 4.93 |
|  | *FGFR1p. A266S* | 6.5 | 5.32 |
|  | *TP53 p.S241F* | 6.25 | 2.29 |
|  |  |  |  |
| 6.25% (1:16) | *KRAS p.G13D* | 3 | 2.54 |
|  | *PIK3CA p.D549N* | 3.12 | 3.18 |
|  | *KIT p.V532I* | 2.9 | 2.00 |
|  | *SMO p.T640A* | 3.12 | 1.00 |
|  | *FGFR1p. A266S* | 3.25 | 4.83 |
|  | *TP53 p.S241F* | 3.12 | 1.0 |
|  |  |  |  |
| 3.25% (1:32) | *KRAS p.G13D* | 1.5 | 1 |
|  | *PIK3CA p.D549N* | 1.56 | 1 |
|  | *KIT p.V532I* | 1.45 | 1 |
|  | *SMO p.T640A* | 1.56 | 1 |
|  | *FGFR1p. A266S* | 1.62 | 1 |
|  | *TP53 p.S241F* | 1.56 | 0 |

**S4 Table**: Sensitivity study on Ion S5XL platform using serially diluted DLD 1 cell line positive for mutation in *PIK3CA, KRAS, KIT, TP53, FGFR1* and *SMO*.
