# Supplementary material for: Versatile ion S5XL sequencer for targeted next generation sequencing of solid tumors in a clinical laboratory
Source: PLoS One. 2017 Aug 2;12(8):e0181968. doi: 10.1371/journal.pone.0181968 (PMC5540534; doi:10.1371/journal.pone.0181968)
Supplement: S5 Table — (DOCX) [file pone.0181968.s006.docx]

**S5 Table**: Inter reproducibility study using 1 CCP and 1 OCP normal tumor paired libraries from same patient

| **Variant** | **Run 1** | | **Run 2** | |
| --- | --- | --- | --- | --- |
|  | **CCP** | **OCP** | **CCP** | **OCP** |
| *ATM p.V185I* | 61.5 | 61.78 | 60.9 | 63.45 |
| *APC p.E1521K* | 8.88 | 8.22 | 8.81 | 7.98 |
| *CCNE1 p.A279T* | 12.6 | 12.08 | 12.3 | 1 |
| *FBXW7 p.E297K* | 41.5 | 32.7 | 40.2 | 31.24 |
| *FGFR4 p.V580M* | 7.93 | 12.7 | 7.45 | 13.6 |
| *JAK1 p.G741D* | 18.6 | 22.12 | 18.56 | 21.23 |
| *NF2p.G569D* | 18.4 | 18.2 | 17.61 | 16.88 |
| *MSH2 p.G862E* | 4.15 | 5.78 | 3.89 | 4.36 |
| *RB1 p.T142I* | 15.48 | 18.52 | 16.41 | 17.42 |
